# Can socioeconomic disadvantage explain cognitive differences in later life? Insights from the Indonesia family life survey

**DOI:** 10.3389/fpubh.2025.1563543

**Published:** 2025-05-27

**Authors:** Sujarwoto Sujarwoto, Tri Yumarni, Holipah Holipah, Asri Maharani

**Affiliations:** ^1^Department of Public Administration, University of Brawijaya, Malang, Indonesia; ^2^Department of Public Health, University of Brawijaya, Malang, Indonesia; ^3^Department of Nursing and Midwifery, The University of Manchester, Manchester, United Kingdom

**Keywords:** cognitive impairment non-dementia, dementia, longitudinal analysis, Indonesia, social economic status

## Abstract

**Introduction:**

Cognitive decline, including cognitive impairment non-dementia (CIND) and dementia, is a growing public health concern, particularly in ageing populations within developing countries. Socioeconomic status (SES) is increasingly recognized as a key determinant of cognitive ageing, yet evidence from low- and middle-income contexts remains limited. This study investigates the relationship between SES and later-life cognitive outcomes in Indonesia.

**Methods:**

We analysed longitudinal data from Waves 4 and 5 of the Indonesia Family Life Survey (IFLS), involving 3,087 participants aged 50 years and older at baseline (Wave 4). Cognitive outcomes, including CIND and dementia, were assessed seven years later using an adapted version of the Telephone Interview for Cognitive Status (TICS). Multilevel ordinal regression was employed to evaluate the association between SES indicators at baseline—such as education, income, residential location, and participation in community-based older adults health posts (*Posbindu Lansia*)—and subsequent cognitive outcomes.

**Results:**

At follow-up, 38% of the sample exhibited CIND, and 19% were classified as having dementia. Higher levels of formal education, greater income, urban residence, and engagement in *Posbindu Lansia* activities were significantly associated with reduced risk of both CIND and dementia.

**Discussion:**

Findings suggest that SES disparities contribute to cognitive decline in later life. Interventions aimed at improving educational attainment, economic conditions, and community health access among older adults may serve as critical strategies to mitigate the future burden of dementia. Reducing social inequalities in health should therefore be prioritized in dementia prevention policies within low-resource settings.

## 1 Introduction

The prevalence of dementia is increasing in the developing world in tandem with the aging of the population. The number of people with dementia in low- and middle-income countries is projected to increase from 14.5 million in 2001 to 81.1 million in 2040 (1, 2). The growing prevalence of dementia will unavoidably place a more substantial burden of disease on those countries as almost 6% of 112,000 disability-adjusted life years due to all diseases are lost to.

Alzheimer diseases and other dementias. This loss makes understanding the epidemiology indices and risk factors of dementia in developing countries crucial, especially for the making of policy in the public health sector.

Some limitations are evident in the emerging literature. Firstly, studies on the epidemiology of dementia in developing countries are meager compared to the extensive studies emerging from developed countries and lack in many world regions (3). Despite several studies have been done in China, and India, there was a dearth of published studies in other countries, including Africa, the Middle East and Indonesia (4). Secondly, studies on the epidemiology of dementia in developing countries tend to use community or volunteer samples rather than national sample studies (5, 6). Finally, little is known regarding the neighborhoods’ effect on dementia, especially in developing countries (7, 8). A study in Indonesia demonstrated that the geographic location and community-level socioeconomic status were associated with pregnancy-related health behaviors (9). Research looking at dementia and its risk factors thus needs to consider potential environmental determinants.

To fill these gaps, this study used the Indonesia Family Life Survey (IFLS), an extensive nationally representative survey in Indonesia, to study the prevalence and determinants of dementia in Indonesia. Indonesia is one of the countries facing the challenge of supporting a growing population with dementia as it has the fourth-largest population of older adults in the world after China, India, and the United States. The percentage of people aged 60 and over in Indonesia has increased from 3.7% in 1960 to 9.7% in 2011 (10). The population of older people is projected to reach approximately 74 million (one-fourth of the Indonesian population) by 2050. A recent study in Indonesia showed that the prevalence of possible dementia among individuals aged 65 years and older in Indonesia in 2014 was 6.8% (11). The Global Burden of Disease Study estimated that more than 1.1 million Indonesians had with Alzheimer’s disease and other dementias in 2016 (2). This study contributes to the existing literature in several ways. Firstly, it uses an objective measurement of dementia using an adapted version of the Telephone Interview for Cognitive Status (TICS) (12). Secondly, this study is among the first to use a longitudinal nationally representative study in a developing country (Indonesia) in this research area. Finally, we applied three-level hierarchical logistic regression to account for unobserved factors in household and communities’ level while considering a range of risk factors in individual level, including demographic, socio-economic and health status. Specifically, the research questions to be addressed are: To what extent do demographic and socioeconomic status at the individual level affect the presence of CIND and dementia later in life? What is the relationship between household expenditure and individuals’ risk of having CIND and dementia 7 years later? Does the number of village health posts in community level influence and individuals’ risk of having CIND and dementia later in life?

## 2 Methods

### 2.1 Data

The IFLS is a longitudinal survey that was first carried out in 1993 by the RAND Corporation in collaboration with several Indonesian universities. Since then, four waves of follow-up data collection have been conducted: in 1997, 2000, 2007 and 2014. The IFLS collects a wide range of data on socio-economic, health and cognitive status from more than 30,000 individuals. It representative of about 83% of the entire Indonesian population as it collected the data from individuals in 13 of 27 provinces in the country. In this study, we used data from the two most recent waves (waves 4 and 5), which were conducted in 2007 and 2014. Our study sample of 3,087 individuals included IFLS wave 4 participants aged 50 and older who responded to the cognitive tests in the wave 5.

### 2.2 Cognitive measures

The IFLS assesses cognitive function in respondents with tests adapted from the TICS (12). The TICS included the scores from an immediate and delayed 10-noun free recall test, a serial of 7 subtraction test, and a backward count from 20 test. Total scores ranged from 0 to 27. To define cognitive status, including a probable dementia diagnosis, we used score cut-offs developed by Langa and Weir (12). They categorized individuals scoring 0 to 6 points on the 27-point TICS scale as having probable dementia, 7 to 11 points as having possible cognitive impairment not dementia (CIND), and 12 to 27 points as having normal cognitive function. Crimmins and colleagues further evaluated these cut-off points against the prevalence of dementia and CIND in the Aging, Demographics, and Memory Study (ADAMS) (13). In this study, respondents who scored from 0 to 6 were classified as having dementia, 7 to 11 as having CIND, and 12 to 27 as normal. As the IFLS wave 4 has no TICS information, we used episodic memory scores at wave 4 as a control variable of the cognitive function in wave 5.

### 2.3 Covariates

Our study included demographics, ethnicity, religion, socioeconomic status, social capital, smoking behavior, physical activities, Body Mass Index (BMI), depression and the presence of chronic diseases as individual-level covariates of cognitive function. We treated age as a continuous variable and entered gender as a dummy variable with the male as the reference. Ethnicity was classified as Javanese or other ethnic groups, while religion was categorized as Muslim or other religions. Marital status was categorized as single as the reference, married, divorced, and widowed. Education was categorized into less than high school, high school and college or higher, with less than high school as the reference. Employment status was entered as a dummy variable with unemployed as the reference. Social capital is the sum of four activities in the community attended by respondents in the last 12 months: community meeting, voluntary labor, the program to improve the village/neighborhood, and religious activities.

The IFLS assesses mental health status using the 10-item Center for Epidemiologic Studies Depression scale (CES-D), which has been commonly used to measure depressive symptoms in population studies in developing countries, including Indonesia. The scores range from 10 to 40 after reverse-coding the positively phrased items. We entered moderate and vigorous physical activities as the number of days per week that respondents engaged in such activities. The self-reported chronic medical conditions included were diabetes, hypertension, stroke, chronic lung diseases and cancer. Body Mass Index (BMI) was categorized as underweight (<18.5 kg/m^2^), normal weight (18.5–24.9 kg/m^2^), overweight (25.0–29.9 kg/m^2^), or obese (≥30.0 kg/m^2^).

The covariate in household level is per-capita household consumption expenditure in tertile. In developing countries, including Indonesia, expenditure captures levels of long-term economic resources more accurately than income as it reflects households’ ability to meet (or exceed) their material needs. Literature suggests that geographic context may contribute to the cognitive function (11). For the covariate in community level, we included urban/rural category and the number of village health post for older adults (*Pos Pelayanan Terpadu Lanjut Usia* or *Posyandu* Lansia). In 2004, the Government of Indonesia launched a policy to improve the quality of life among older adults and required each community to have village health posts for them, which is known as *Pos Pelayanan Terpadu Lanjut Usia* or *Posyandu Lansia* (14). *Posyandu,* as a community health post, initially provides basic health services for young children (under 5 years old) and pregnant mothers. After 2004, some of *Posyandu* expands their services for older adults. *Posyandu Lansia* provides health care services for adults (45–59 years old) and older adults (60 years and older). Those services include basic physical and mental health care (by nurses or midwives), preventing cognitive decline, preventive and promotion (especially for non-communicable diseases), and nutritional care. The availability of mental health care and program to prevent cognition decline in *Posyandu Lansia* is the main reason we include it in our analysis.

### 2.4 Statistical analysis

We conducted data analysis in two steps: bivariate analysis and multivariate analysis. The bivariate associations between cognitive function (the presence of CIND and dementia) and independent variables were examined with ordinal regression. The multivariate analysis identified the association between cognitive performance and all of the risk factors together using multilevel ordinal regression models to take into account of the household and community level information available from the IFLS. By accounting for the multilevel structure of individuals within households and households within the community, we were able to investigate whether the effect household economy conditions and community characteristics on individual health outcomes vary between households and communities. Multilevel ordinal regression analysis models’ variables at different levels without aggregated or disaggregated them. Aggregation and disaggregation, as used in the single-level model, run the risk of ecological fallacy. Multilevel ordinal regression analysis offers rich opportunities to explore contextual effects by incorporating characteristics of households and communities as well as those of individuals (15).

The first level comprised individual characteristics, the second level was household characteristics, and community characteristics made up the third level. Considering individual *i* nested in household *j*, and community *k*:


$$Y_{\mathrm{ijk}}=\gamma_{000}+\sum \gamma_{000k\;U_{k}}+\sum \gamma_{0\mathrm{jk}\;W_{\mathrm{jk}}}+\sum \beta_{\mathrm{ijk}\;X_{\mathrm{ijk}}}+u_{00j}+r_{0\mathrm{jk}}+\in_{\mathrm{ijk}}$$

with:

*Y_ijk_* = cognitive function as an ordinal variable (normal, CIND and dementia) for the individual in household *j* in community *k*.

*U_k_* is a set of community characteristics,

*W_jk_* is a set of household and community characteristics, *X_ijk_* is a set of individual characteristics, *u_00j_* are the random intercept varying over the household *r_0jk_* is the random intercept varying over household and community.

є*ijk* is normally distributed with mean zero and variance σ_є_^2^.

The multivariate analysis used three models. The first model included only the sociodemographic variables of age, gender, marital status, education, employment status, and social capital. We added smoking behavior, physical activities, depression and the presence of chronic diseases in the second model and household expenditure in tertile as the household level determinant, and rural/urban category and the number of *Posyandu Lansia* as the community level determinants in the final model. Longitudinal weights were applied in all analyses to compensate for unit non-response, and for attrition between the fourth and fifth waves. We conducted the hierarchical logit regression using *meologit* commands in STATA 19 software.

## 3 Results

Table 1 shows the descriptive characteristics of the 3,087 respondents included in this study. The average age at baseline of the respondents was 57.4 (standard deviation = 6.9). Slightly more than half (53.4%) of them were male. They were mostly (80.1%) had no education or primary (less than high school) education, and only 7.1% of the respondents were graduated from college or higher education. More than one-third of them were active smokers and on average they did moderate and vigorous physical exercise four and less than 2 days a week, respectively. The prevalence of hypertension is higher compared other chronic diseases. Using the ADAMS dementia diagnosis, we found that 38 and 19% of the respondents had CIND and dementia, respectively. The bivariate analyses showed that older age, female, Muslim, lower education, lower level of social capital, lower household expenditure, hypertension, underweight and rural residence at baseline were associated with higher odds of having CIND and dementia 7 years later. On average, the number of *Posyandu Lansia* in each community at baseline was 1.1 (SD = 2.1). A higher number of *Posyandu Lansia* in the community has a negative and significant association with the presence of CIND and dementia.

**Table 1:** Characteristics of the study sample.

Variables	Total	Normal	CIND	Dementia	Bivariate analysis
	*n* = 3,087	*n* = 1,371	*n* = 1,180	*n* = 590	OR (95% CI)
Individual level					
Age, mean (SD)	57.4(6.9)	56(6.7)	57.7(6.3)	59.9(7.5)	1.07(1.05; 1.08) ‡
*Ethnic background, %*					
Other ethnics	57.3	51.6	51.0	51.2	Ref.
Javanese	42.7	48.4	49.0	48.8	1.02(0.89;1.16)
*Religion, %*					
Other religions	9.0	14.3	10.7	16.7	Ref.
Muslim	91.0	85.7	89.3	89.3	1.34(1.01;1.65) ‡
*Sex, %*					
Male	53.4	56.7	51.9	49.8	Ref
Female	46.6	43.3	48.1	50.2	1.23(1.06; 1.42) ‡
*Marital status, %*					
Single	0.6	0.9	0.3	0.9	Ref
Married	81.4	83.7	81.9	75.6	1.36(0.40; 4.65)
Separated/divorced	2.7	2.2	2.9	3.6	1.94(0.53; 7.10)
Widowed	15.3	13.1	14.9	19.9	1.93(0.56; 6.68)
*Education, %*					
Less than high school	80.2	68.7	87.8	94.5	Ref
High school	12.6	18.8	8.6	4.6	0.29 (0.23; 0.37) ‡
College and higher	7.2	12.5	3.6	0.9	0.16 (0.11; 0.24) ‡
*Employed, %*					
No	25.6	26.7	24.4	25.9	Ref
Yes	74.4	73.3	75.6	74.1	1.06(0.90; 1.25)
Social capital, mean (SD)	1.6(1.2)	1.7(1.2)	1.6(1.2)	1.4(1.1)	0.87(0.82; 0.92)‡
*Current smoker, %*					
No	60.6	61.9	59.4	57.5	Ref
Yes	39.3	38.1	40.6	42.5	1.14(0.98; 1.33)
Depression score, mean (SD)	5.7(4)	5.6(3.9)	5.7(4)	6.1(4.4)	1.01(0.99; 1.03)
Moderate exercise, mean (SD)	4.5 (2.8)	4.4 (2.8)	4.5 (2.8)	4.7 (2.8)	1.01(0.99; 1.04)
Vigorous exercise, mean (SD)	1.8(2.8)	1.6(2.6)	1.9(2.8)	2.1(2.9)	1.05(1.02; 1.07) ‡
*Diabetes, %*					
No	97.1	95.6	97.9	98.4	Ref
Yes	2.9	4.4	2.1	1.6	0.44(0.28; 0.69) ‡
*Hypertension, %*					
No	82.4	83.5	81.5	81.5	Ref
Yes	17.6	16.5	18.5	18.5	1.12(0.94; 1.35)
*Chronic lung diseases, %*					
No	98.2	97.5	99.1	97.5	Ref
Yes	1.8	2.5	0.9	2.5	0.75(0.39; 1.47)
*Stroke, %*					
No	99.3	99.3	99.3	99.5	Ref
Yes	0.7	0.7	0.7	0.5	0.82(0.39; 1.72)
*Cancer, %*					
No	99.5	99.6	99.7	99.7	Ref
Yes	0.5	0.4	0.3	0.3	0.76(0.24; 2.39)
*BMI category, %*					
Underweight	13.0	9.7	14.7	17.0	1.38(1.12;1.70) ‡
Normal weight	59.7	54.4	57.9	61.2	Ref.
Overweight	21.3	27.0	22.0	17.9	0.69(0.58;0.82) ‡
Obese	6.0	8.9	5.4	3.9	0.50(0.37;0.67) ‡
Episodic memory, mean (SD)	6.6(3.39)	8.0(3.1)	6.0(3.1)	4.5(2.8)	0.78(0.76; 0.80) ‡
Household level					
*Household expenditure, %*					
Lowest tertile	34.5	26.6	36.3	47.5	Ref
Middle	33.5	33.4	35.3	30.2	0.63(0.52; 0.75) ‡
Highest tertile	32.0	40.0	28.4	22.3	0.42 (0.35; 0.51) ‡
Community-level					
*Living in urban area, %*					
No	56.3	46.6	62.6	68.2	Ref
Yes	43.7	53.4	37.4	31.8	0.50(0.43; 0.58) ‡
Number of *Posyandu Lansia*, mean (SD).	1.1(2.1)	1.3(2.5)	0.9(1.7)	0.9(1.7)	0.92(0.89; 0.95) ‡

OR, odds ratio; CI, confidence intervals; Sig.: ‡significant at 1% or less.

Table 2 displays the results of the multilevel ordinal regression models with cognitive function as the outcome variable. The multilevel ordinal regression analysis was carried out using three models. The first model included demographic and socio-economic variables, while the second model included demographic, socio-economic, smoking behavior, physical activities, and the presence of chronic diseases. Results from the first model showed that age, female, Muslim, employed, education level and episodic memory are all statistically significant at 1%. The relationships between age, female, Muslim, and educational level, with the presence of dementia, remain significant in the Model 2 and Model 3. Among the demographic and socioeconomic determinants, it appears that educational attainment are the most influential.

**Table 2:** Three-level ordinal logistic regression results for the determinants of dementia.

Variables	Model 1				Model 2				Model 3			
	OR	*p*-val	95% CI		OR	*p*-val	95% CI		OR	*p*-val	95% CI	
			Low	Up			Low	Up			Low	Up
Individual level												
Age	1.06	0.00	1.05	1.07	1.06	0.00	1.05	1.07	1.03	0.00	1.01	1.05
Female	1.52	0.00	1.16	2.05	1.53	0.00	1.16	2.05	1.54	0.00	1.15	2.05
Javanese	0.90	0.18	0.76	1.05	0.91	0.23	0.77	1.06	0.71	0.11	0.47	1.08
Muslim Marriage status	1.49	0.00	1.17	1.91	1.47	0.00	1.15	1.88	1.81	0.04	1.02	3.21
*(Ref. unmarried)*												
Married	0.79	0.61	0.32	1.95	0.81	0.65	0.33	2.00	0.71	0.63	0.18	2.82
Separated	1.16	0.77	0.43	3.10	1.15	0.78	0.43	3.08	0.88	0.87	0.20	3.87
Widower	0.76	0.56	0.30	1.91	0.78	0.59	0.31	1.94	0.78	0.73	0.19	3.15
*Education status (Ref. less than high school)*												
High school College and	0.43	0.00	0.34	0.55	0.46	0.00	0.36	0.59	0.70	0.07	0.48	1.02
higher	0.24	0.00	0.16	0.34	0.26	0.00	0.18	0.38	0.27	0.00	0.15	0.48
Employed	1.33	0.00	1.13	1.58	1.28	0.01	1.07	1.53	1.08	0.56	0.83	1.39
Social capital	0.99	0.71	0.93	1.05	0.99	0.69	0.92	1.05	1.00	0.93	0.91	1.11
Episodic memory	0.84	0.00	0.82	0.86	0.84	0.00	0.82	0.86	0.93	0.00	0.90	0.96
Current smokers Mean					1.14	0.19	0.94	1.39	1.30	0.05	1.00	1.71
depression score Moderate					1.02	0.07	1.00	1.03	1.01	0.48	0.98	1.03
exercise Vigorous					1.01	0.37	0.99	1.04	1.01	0.57	0.97	1.05
Exercise					1.01	0.36	0.98	1.04	1.00	0.87	0.96	1.04
*BMI category (Ref. normal BMI)*												
Underweight					1.12	0.32	0.90	1.39	1.16	0.32	0.86	1.57
Overweight					1.04	0.70	0.86	1.25	0.79	0.08	0.61	1.03
Obese					0.78	0.14	0.56	1.08	0.91	0.70	0.57	1.46
*Chronic diseases*												
Diabetes					0.78	0.28	0.49	1.22	0.69	0.27	0.36	1.34
Hypertension					1.23	0.03	1.02	1.48	1.23	0.11	0.95	1.60
Chronic lung diseases					0.90	0.72	0.52	1.58	1.67	0.20	0.76	3.65
Stroke					0.95	0.89	0.45	2.01	0.69	0.46	0.26	1.84
Cancer					0.72	0.56	0.25	2.13	1.70	0.47	0.40	7.28
Household level												
*Household expenditure (Ref. lowest expenditure)*												
Middle									0.72	0.01	0.56	0.93
Highest tertile									0.75	0.04	0.55	0.99
Community level												
Urban									0.31	0.00	0.18	0.52
*Posyandu Lansia*									0.44	0.00	0.41	0.47
Intercept 1	2.01		0.83	3.19	2.20		1.01	−3.39	−3.71		−5.60	−1.82
Intercept 2	4.12		2.93	5.31	4.32		3.12	5.51	0.50		−1.37	−2.38
Variance between households	0.24				0.25		0.25		0.18			
Variance between communities	0.05				0.05		0.05		0.05			

Hypertension and smoking are associated with higher odd of having CIND and dementia 7 years later than those without hypertension and non-smokers. Depression and physical activities have no significant association on cognitive function.

In the household level, respondents in the second and highest tertiles of household expenditure had 56 and 55% lower odds of having CIND and dementia 7 years later than those in the poorest tertile. Living in an urban area was associated with lower odds of having CIND and dementia. The number of *Posyandu Lansia* has significant association with lower odds of having CIND and dementia in the final model.

**Figure 1:**
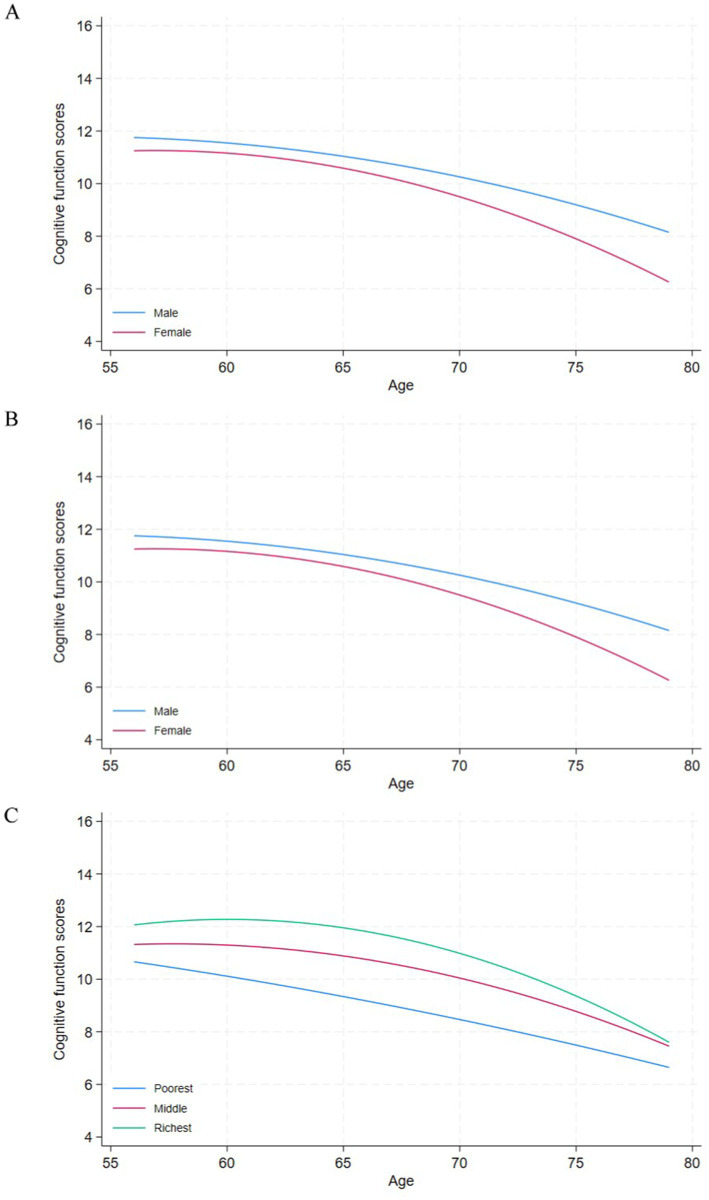
Cognitive function and age among older adults in IFLS wave 5 by gender, education attainment and household expenditure at baseline.

Figure 1 plots cognitive scores as a function of age with separate curves for gender, education and income at baseline. Figure 1A shows that females demonstrated lower cognitive function than males. Older age was negatively associated with cognitive ability among both males and females, and that negative association was steeper among females. Figure 1B plots the age profiles of cognitive scores separately according to respondents’ educational attainment at baseline. This figure supports the hypothesis that education is an important factor of heterogeneity in cognitive ability at older ages. Higher educational attainment corresponds to better cognitive ability at all ages. Figure 1C describes the age profiles of cognitive scores by log household expenditures at baseline. In addition to showing large differences in cognitive scores between respondents with different expenditures, it documents different shapes of the cognitive score plots. The plot of cognitive scores of older adults with higher expenditures (richest and medium) followed curvilinear shapes, while that of poorest older adults showed a more precipitous pattern of cognitive decline after the age of 57.

### 3.1 Sensitivity analysis

To assess the robustness of our classification of probable dementia, CIND, and normal based on a TICS score, we conducted a sensitivity analysis by varying the threshold used to define dementia, CIND, and normal. Specifically, we tested two alternative cut-offs. First, stricter cut off (0–5 = dementia), (6–10 = CIND), and (>11 = normal). Second, a more inclusive cut off (07 = dementia), (8–12 = CIND), and (>13 = normal). This follows approaches used in previous studies analyzing cognitive impairment in population-based surveys (16). For each threshold, we re-estimated key outcomes—such as prevalence rates and associations with demographic and health-related predictors—to determine whether the direction, magnitude, and statistical significance of results remained consistent. This approach allows us to evaluate the stability of our findings under different plausible definitions of dementia and to assess potential misclassification around the chosen threshold. Consistent results across these cut-offs strengthen the validity of our primary conclusions, while any variation may highlight sensitivity to classification criteria, underscoring the need for cautious interpretation in research and policy contexts (17, 18). Table 3 presents the results using both cut-off points, and overall, the results are similar to those obtained using main cut off classification.

**Table 3:** Sensitivity analysis results.

Variables	Model A				Model B			
	OR	*p*-val	95% CI		OR	*p*-val	95% CI	
			Low	Up			Low	Up
Individual level								
Age	1.03	0.00	1.01	1.04	1.02	0.02	1.00	1.04
Female	1.57	0.00	1.16	2.13	1.56	0.00	1.14	2.03
Javanese	0.61	0.03	0.40	0.95	0.55	0.00	0.36	0.82
Muslim	1.34	0.36	0.72	2.48	1.84	0.03	1.07	3.14
*Marriage status (Ref. unmarried)*								
Married	0.89	0.87	0.21	3.76	1.61	0.47	0.44	5.83
Separated	1.21	0.81	0.26	5.62	2.37	0.23	0.59	9.56
Widower	0.94	0.94	0.22	4.06	1.88	0.35	0.51	6.95
*Education status (Ref. less than high school)*								
High school	0.63	0.03	0.42	0.96	0.60	0.01	0.42	0.86
College and higher	0.19	0.00	0.09	0.38	0.29	0.00	0.17	0.48
Employed	1.02	0.87	0.78	1.33	0.95	0.72	0.74	1.23
Social capital	0.95	0.37	0.86	1.06	0.97	0.56	0.88	1.07
Episodic memory	0.92	0.00	0.89	0.96	0.92	0.00	0.89	0.95
Current smokers	1.14	0.37	0.86	1.50	1.28	0.07	0.98	1.67
Mean depression score	1.01	0.64	0.98	1.03	1.02	0.10	1.00	1.05
Moderate exercise	1.02	0.35	0.98	1.06	1.00	0.96	0.96	1.04
Vigorous exercise	1.00	0.85	0.96	1.05	0.99	0.67	0.95	1.03
*BMI category (Ref. normal BMI)*								
Underweight	1.11	0.50	0.82	1.51	0.95	0.72	0.70	1.28
Overweight	0.73	0.03	0.55	0.96	0.86	0.26	0.67	1.12
Obese	0.95	0.83	0.57	1.56	0.83	0.43	0.53	1.32
*Chronic diseases*								
Diabetes	0.56	0.10	0.28	1.13	0.81	0.51	0.44	1.50
Hypertension	1.42	0.01	1.08	1.85	1.46	0.00	1.13	1.90
Chronic lung diseases	1.27	0.58	0.55	2.90	2.09	0.05	1.00	4.35
Stroke	0.75	0.58	0.26	2.10	0.64	0.36	0.25	1.66
Cancer	1.71	0.48	0.39	7.59	0.71	0.64	0.17	3.04
Household level								
*Household expenditure (Ref. lowest tertile)*								
Middle	0.74	0.02	0.57	0.95	0.75	0.03	0.58	0.96
Highest tertile	0.72	0.03	0.54	0.97	0.62	0.00	0.47	0.82
Community level								
Urban	0.37	0.00	0.22	0.65	0.32	0.00	0.19	0.55
*Posyandu Lansia*	0.44	0.00	0.41	0.47	0.43	0.00	0.41	0.46
Intercept 1	−3.04		−5.00	−1.08	−4.75		−6.58	−2.92
Intercept 2	1.06		−0.88	3.01	−0.55		−2.37	−1.26
Variance between households	0.24				0.24			
Variance between communities	0.05				0.05			

## 4 Discussion

Using a nationally representative survey of older Indonesians, we found that the prevalence of cognitive impairment not dementia (CIND) was 38%, while the prevalence of dementia was.

19% among individuals aged 57 years and older in 2014, 7 years after the baseline survey. These figures are considerably higher than those reported in other low- and middle-income countries (LMICs), where dementia prevalence typically ranges between 3 and 8%. For instance, reported prevalence rates in India range from 4 to 5%, in China from 6 to 7%, in Bangladesh 3%, in Sri Lanka 4%, in Malaysia 4%, in Thailand 5 to 6%, in Uganda and Nigeria 3%, in Argentina 8%, and in Cuba 12.6% (2, 19–21). The notably higher prevalence of dementia observed in Indonesia may be attributable, at least in part, to methodological differences across studies, particularly in the measures and diagnostic criteria used to assess cognitive function and dementia. Variability in tools, cut-off points, and cultural adaptation of cognitive assessments may contribute to inconsistencies in prevalence estimates across countries (2, 21). Further research is needed to evaluate the extent to which these methodological factors influence cross-country comparisons.

However, our findings are consistent with other studies that employed similar methodologies within Indonesia. Recent research indicates that the prevalence of dementia ranges from 20.1 to 29.15% across various regions, with the highest rates often observed in rural areas (22). A 2021 community-based study estimated a dementia prevalence of 27.9% among individuals aged 65 years and older, corresponding to over 4.2 million people living with dementia in the country (22). Despite these high prevalence rates, formal diagnosis remains extremely limited, with only 0.2% of individuals having received a medical diagnosis of dementia. Additionally, public awareness of dementia is minimal, with 86.3% of the population reportedly unfamiliar with the condition (23). Future projections suggest that by 2050, nearly 4 million Indonesians will be living with dementia, underscoring the urgent need for improved strategies in diagnosis, public education, and long-term care (22). Thus, our findings have significant implications for the capacity of developing countries to provide future health care for older adults. With.

Indonesia, India and China together constituting the world’s largest older population, one can expect a substantial increase in the number of persons with dementia (24, 25). The reason Indonesia is considered high compared to other LMICs could be due to various factors, including better awareness and diagnosis in urban areas, and the challenges posed by a large, aging population with various socio-economic disparities.

The prevalence rate of dementia increased with age in the present study, as has been reported in other studies (19, 25, 26). One of the consistent findings in the literature in both developed and developing countries that the prevalence of dementia increases among those with lower educational levels at baseline (26, 27). Our study confirmed this finding and supported the hypothesis that ‘cognitive reserve’ resulting from early-life and lifelong education reflects the persistence of differences in cognitive ability (28). Improving access to education may thus be a potent strategy for the primary prevention of dementia in low- and middle-income countries around the world. In the household level, we also found that respondents in the household with higher expenditure per capita were at lower risk of CIND and dementia 7 years later.

Our findings extend previous research in developing countries by plotting the cross-sectional trajectories of cognitive function according to gender, education and wealth 7 years prior. For example, prior studies reported gender inequality in cognitive impairments among older adults in Asia, Latin American and The Caribbean remains a significant concern (29–32). Prior studies provide compelling evidence that gender inequality hinders national economic growth, highlighting the substantial macroeconomic consequences of unequal access to education and labor market opportunities. These foundational structural barriers likely explain the gender-based differences in cognitive function observed in our data, as limited educational and economic opportunities early in life can have enduring negative impacts on cognitive reserve and its trajectory (30, 33).

Yu’s investigation into the frequently overlooked influence of inequality on global mental health demonstrates a strong link between socioeconomic disparities and well-being (34). This finding supports our observation of the close relationship between wealth and education with cognitive outcomes, implying that efforts to decrease inequality could significantly help mitigate cognitive decline and improve population-level mental health. Findings indicate that equalizing access to education and employment could substantially reduce gender gaps in cognitive impairment (32). Promoting gender-responsive policies that enhance educational and occupational opportunities for women is essential. Furthermore, community-based interventions—such as informal support networks for widows and initiatives that encourage physical activity and social engagement—may be effective in mitigating cognitive decline. These insights offer important guidance for policymakers aiming to advance gender equity and promote healthy cognitive aging.

Focusing on wealth, the negative slope of the cognitive trajectory of respondents with lower household expenditure is steeper than that of respondents with higher expenditure. This finding indicates that the negative association between age and cognitive ability is stronger among respondents with lower educational attainments and household expenditure at baseline. The lower socioeconomic status may associate with cognitive decline later in life using several mechanisms. Literature has shown the negative association between low socioeconomic status and both physical and mental health among older people which may increase the risk of cognitive impairment in later life (35, 36). Studies across various contexts, including India and China, have consistently demonstrated that individuals from lower social economic status backgrounds experience higher rates of cognitive impairment, partly due to lower cognitive reserve, chronic stress, and unhealthy lifestyle behaviors. These findings suggest that socioeconomic disadvantage not only influences immediate health outcomes but also has long-term implications for brain health and cognitive functioning in aging populations (36–38).

Higher educational attainment is also closely linked to healthier lifestyle choices and improved access to healthcare, both of which contribute to a reduced risk of developing dementia. Individuals with more education are more likely to engage in beneficial health behaviors, such as regular physical activity, maintaining a nutritious diet, and abstaining from smoking. These behaviors are associated with a lower risk of cognitive decline and dementia. For instance, adherence to a Mediterranean-type diet and higher levels of physical activity were independently associated with a reduced risk of Alzheimer’s disease. Moreover, higher education levels often correlate with better access to healthcare services, enabling early detection and management of health conditions that could otherwise increase dementia risk. Additionally, education contributes to the development of cognitive reserve—the brain’s resilience to neuropathological damage—which can delay the onset of dementia symptoms (39). A study in Korea, Cina, India, and Malaysia demonstrated that individuals with higher educational attainment had a lower lifetime risk of dementia, emphasizing the protective role of education. Therefore, promoting educational opportunities and healthy lifestyle behaviors may serve as effective strategies in reducing the incidence of dementia (40).

Individuals with lower socioeconomic status (SES) are also more likely to experience chronic psychological stress and elevated allostatic load—the cumulative physiological burden resulting from repeated or prolonged stress—which have been shown to negatively affect cognitive function. In LMICs, individuals with lower SES are particularly vulnerable to chronic psychological stress and elevated allostatic load, which can negatively impact cognitive health. Socioeconomic disadvantages in LMICs—such as unstable employment, limited access to education, inadequate healthcare, and food insecurity—expose individuals to persistent stressors that activate the body’s stress-response systems over extended periods, leading to physiological dysregulation. This accumulated burden, known as allostatic load, has been associated with impairments in cognitive function, as demonstrated in various studies. For example, a study in India found that individuals from lower economic strata exhibited significantly higher rates of cognitive impairment, partially mediated by psychosocial stress and poor physical health (38). Chronic stress is also known to disrupt hippocampal neurogenesis and has been shown in both animal models and human studies to reduce hippocampal volume—an area of the brain crucial for learning and memory. These effects are particularly concerning in LMICs, where healthcare systems may lack the capacity for early detection and intervention for cognitive decline. The biological mechanisms through which chronic stress affects the brain—such as increased cortisol levels and inflammation—have been well documented, and their cognitive consequences are compounded by structural inequalities in LMIC contexts (41, 42). These findings highlight the urgent need to address socioeconomic inequities and chronic stress exposure to preserve cognitive function and promote brain health across disadvantaged populations in LMICs.

In the community level, living in urban areas in Indonesia is associated with a lower risk of dementia compared to rural settings, primarily due to better access to quality education and healthcare services. Urban residents often benefit from higher educational attainment, which enhances cognitive reserve and resilience against neurodegenerative processes. For instance, a study in rural Tanzania found a significant association between low levels of education and increased dementia risk, highlighting the protective role of education (43). Moreover, urban areas typically offer more comprehensive healthcare infrastructure, facilitating early diagnosis and management of chronic conditions like hypertension and diabetes, which are known risk factors for dementia. In India, research has demonstrated significant rural–urban disparities in the diagnosis and treatment of these conditions, with rural populations experiencing higher rates of undiagnosed and untreated cases (44). Additionally, urban environments provide greater access to social and recreational activities, which can contribute to cognitive stimulation and delay the onset of dementia. However, it is important to note that urban living also presents challenges, such as increased exposure to air and noise pollution, which may negatively impact cognitive health. Therefore, while urban residency in LMICs is generally linked to a reduced risk of dementia due to better education and healthcare access, addressing environmental and lifestyle factors remains crucial for comprehensive dementia prevention strategies (45).

Our study revealed a significant association between the number of *Posyandu Lansia* (older adult health centers) and the risk of dementia. This finding suggests that the availability and accessibility of these community-based health centers play a crucial role in mitigating the risk of dementia among older adults. The availability and accessibility of community-based health centers play a crucial role in mitigating the risk of dementia among older adults, particularly in low- and middle-income countries (LMICs). These centers serve as vital platforms for delivering preventive care, early detection, and management of dementia through integrated services. A study assessing the feasibility of a community-adapted multi-domain intervention for dementia prevention among older adults in Japan demonstrated the effectiveness of such interventions in community settings (46). The program included physical exercise, cognitive training, nutritional guidance, and vascular risk management, all coordinated through local public health infrastructure. The study found that these community-based interventions are feasible and can be effectively implemented to prevent dementia in older adults. Furthermore, research indicates that cognitively intact older adults residing in resource-rich neighborhoods are less likely to experience cognitive decline. This suggests that the presence of community resources, such as health centers, contributes to better cognitive health outcomes (47). In LMICs, where healthcare resources are often limited, community-based health centers can bridge the gap by providing accessible and culturally appropriate care. These centers can offer education on dementia risk factors, facilitate early diagnosis, and support lifestyle modifications that promote cognitive health. For example, *Posyandu Lansia* in Indonesia, with their focus on health promotion and early detection of health issues, provide a valuable platform for implementing mental health programs specifically designed to prevent cognitive decline (14). These programs could include cognitive stimulation activities, social engagement initiatives, and early identification and management of risk factors such as hypertension, diabetes, and depression, all of which have been linked to an increased risk of dementia. By leveraging the existing infrastructure of *Posyandu Lansia*, we can effectively deliver targeted interventions to older adults, potentially reducing their risk of developing dementia and improving their quality of life in their later years. By integrating dementia care into existing community health services, LMICs can enhance the reach and effectiveness of interventions aimed at reducing the burden of dementia among older adults.

This study has several limitations. First, the cognitive impairment assessment tool used has not been validated in the Indonesian context, although The Telephone Interview for Cognitive Status (TICS) and its modified version (TICS-m) have demonstrated good validity and reliability across diverse populations, including in LMICs and culturally varied settings, making them particularly useful in contexts like Indonesia. These tools are designed for remote cognitive screening and have been validated in populations with low educational attainment and linguistic diversity—challenges common in Indonesian regions. For instance, studies in rural Greece and Iran have shown that TICS and TICS-m are effective in detecting cognitive impairment even among older adults with limited literacy or formal education, demonstrating strong internal consistency and test–retest reliability (48, 49). These findings are supported by evidence from Indonesia itself. Handajani et al., using IFLS-5 data, found that memory impairment among older Indonesian adults was significantly associated with older age, female gender, lower education, depressive symptoms, and stroke history—factors that TICS is sensitive to detecting (50). Similarly, Pengpid et al. used IFLS-5 and found TICS-based assessments effective in evaluating cognitive function in a nationally representative sample, identifying strong associations with social factors, physical inactivity, and comorbidities (11).

Juber et al. further demonstrated TICS’s sensitivity by linking asthma (particularly early-onset) with lower cognitive functioning among older Indonesians, suggesting its utility in identifying at-risk subgroups within the population (51). Together, these studies affirm that TICS and TICS-m are suitable, scalable tools for cognitive screening in Indonesia. When culturally and linguistically adapted, they provide a cost-effective and accessible approach to dementia risk screening, particularly valuable in rural and resource-limited settings.

Second, the inconsistency in cognitive assessment across survey waves. Specifically, Wave 4 of the Indonesia Family Life Survey (IFLS) did not include the Telephone Interview for Cognitive Status (TICS), which was used in Wave 5 to assess cognitive function. As a result, we relied on episodic memory scores available from Wave 4 as a control variable to approximate baseline cognitive function. While episodic memory is a key component of overall cognition, it does not capture the broader range of cognitive domains assessed by the TICS instrument. This discrepancy in measurement tools may introduce bias or imprecision in the estimation of cognitive change over time, potentially affecting the validity of longitudinal comparisons and the accuracy of cognitive decline trajectories. Future studies would benefit from harmonized cognitive assessments across waves to ensure more consistent and reliable longitudinal analysis.

Third, the use of complete-case analysis, whereby only respondents with full data across both Wave 4 (2007) and Wave 5 (2014) of the Indonesia Family Life Survey (IFLS) were included. While this approach ensures consistency in measuring exposures and outcomes over time and avoids the complexities of imputing missing data, it may introduce attrition bias. Individuals who remained in the study and provided complete data may differ systematically from those who were lost to follow-up or had incomplete responses—particularly in terms of age, health status, cognitive function, and socioeconomic background—thus introducing the potential for selection bias and limiting the representativeness of the findings. Although more advanced methods, such as joint modeling of longitudinal and survival data, can help adjust for attrition, they were not feasible in this study due to the limited number of repeated cognitive outcome measurements and the absence of detailed information on the timing and reasons for dropout. Consequently, our findings should be interpreted with caution, particularly regarding their generalizability to the broader population of older Indonesians.

These findings have significant policy and public health implications for Indonesia and LMICs with rapidly aging populations. The high prevalence of CIND and dementia highlights the urgent need for scalable screening, prevention, and care strategies. Given the links between cognitive impairment and socioeconomic factors such as education, gender disparities, household wealth, and healthcare access, public health efforts must focus on reducing these inequalities. Policies promoting early educational access, particularly for women, can enhance cognitive reserve and delay dementia onset. Addressing poverty-related stress and improving economic security can help mitigate cognitive decline. At the community level, strengthening initiatives like *Posyandu Lansia*—Indonesia’s older adult health centers—can play a key role in early detection and prevention by providing accessible, culturally appropriate services. The association between urban residency and lower dementia risk suggests the importance of equitable health resource distribution, particularly in rural areas. Additionally, addressing chronic stress in lower socioeconomic groups, which is linked to cognitive decline and hippocampal atrophy, should be prioritized.

## 5 Conclusion

The high rates of CIND and dementia revealed in this Indonesian survey align with concerning global increases, particularly impacting LMICs nations. Addressing this requires urgent policy attention, positioning dementia as a national public health priority with a comprehensive action plan mirroring WHO recommendations. Crucial strategies include long-term investments in education and poverty reduction for primary prevention, significantly expanding and enhancing community-based programs like *Posyandu Lansia* with dementia-specific services, and substantial investment in training healthcare professionals and raising public awareness. Furthermore, validating culturally relevant cognitive assessments is essential for accurate diagnosis. By implementing these targeted measures, Indonesia can proactively confront the escalating challenge of dementia and its societal impact.

## Data Availability

Publicly available datasets were analyzed in this study. This data can be found at: https://www.rand.org/well-being/social-and-behavioral-policy/data/FLS/IFLS/access.html.
